# Impact of continuous glucose monitoring on patient-reported outcomes in adults with type 2 diabetes: a Systematic Review and meta-analysis

**DOI:** 10.3389/fendo.2026.1830980

**Published:** 2026-05-20

**Authors:** Haibo Zhang, Jiebin Li, Dawei Wang, Qingxia Du, Jing Zhang

**Affiliations:** Department of Emergency, Beijing Tongren Hospital, Capital Medical University, Beijing, China

**Keywords:** continuous glucose monitoring, health-related quality of life, Self-monitoring of blood glucose, systematic review and meta-analysis, type 2 diabetes

## Abstract

**Introduction:**

This research intended to systematically evaluate the impact of continuous glucose monitoring (CGM) on the emotional well-being of individuals with type 2 diabetes. It focused on the effects of CGM in four distinct patient-reported outcome domains: diabetes distress (measured by the diabetes distress scale [DDS]), treatment satisfaction (diabetes treatment satisfaction questionnaire [DTSQ]), psychological well-being (world health organization-5 well-being index [WHO-5]), and health-related quality of life (EuroQol five-dimensional questionnaire [EQ-5D]).

**Methods:**

PubMed, Web of Science, Embase, and Cochrane Library were retrieved from inception until April 2026 to collect randomized controlled trials (RCTs) comparing CGM with self-monitoring of blood glucose (SMBG). After two researchers independently performed literature screening, data extraction, and quality assessment, meta-analysis was conducted using Stata 16.0.

**Results:**

A total of 9 RCTs comprising 1,453 individuals were included. All five studies reporting treatment satisfaction (DTSQ) showed a direction of effect favoring CGM over SMBG, although the magnitude of improvement varied widely and heterogeneity was extremely high (I²=98.9%), precluding a meaningful single summary estimate. However, no statistically significant differences were observed between the two groups in terms of diabetes distress (DDS) [standardized mean difference (SMD)=-0.25, 95% confidence interval (CI) (-0.98, 0.17)], psychological well-being (WHO-5) [SMD = 0.08, 95% CI (-0.09, 0.25)], or health-related quality of life (EQ-5D) [SMD = 0.22, 95% CI (-0.20, 0.64)].

**Conclusion:**

Current evidence indicates that CGM may improve treatment satisfaction in individuals with type 2 diabetes, although this finding is limited by very high heterogeneity. Its effects on alleviating diabetes distress, improving psychological well-being, and enhancing health-related quality of life remain unclear and should be further investigated.

**Systematic review registration:**

https://www.crd.york.ac.uk/prospero/, identifier CRD42025634725.

## 1 Background

Diabetes is a metabolic disease that severely impacts global health, with its prevalence continuously rising. Approximately 561 million people globally were living with diabetes in 2023, and this number is anticipated to exceed 1.3 billion by 2050 (1–3). Despite continuous advancements in treatment, there are still serious challenges in diabetes management. In 2023, only 41.6% of individuals receiving treatment globally achieved glycemic targets (4). Optimizing glycemic control and enhancing glucose monitoring are focuses of diabetes management (5, 6). In recent years, continuous glucose monitoring (CGM) systems, including intermittently scanned CGM (isCGM) and real-time CGM (rtCGM), have been widely used in clinical practice. CGM systems, comprising isCGM and rtCGM, monitor glucose levels in interstitial fluid via subcutaneous sensors, enabling dynamic tracking of glucose levels without the need for frequent fingertip blood sampling. This technology can visually reflect glucose fluctuations and daily glucose profiles, and it also provides a crucial basis for treatment decisions and behavioral adjustments (7, 8).

In recent years, several meta-analyses have indicated that in comparison with traditional self-monitoring of blood glucose (SMBG), CGM demonstrates advantages in improving glycemic indicators, including glycated hemoglobin (HbA1c) and time in range (TIR) (9–11). However, a systematic review specifically synthesizing evidence across multiple distinct domains of emotional well-being (including diabetes distress, treatment satisfaction, psychological well-being, and health-related quality of life) in adults with type 2 diabetes is currently lacking. In existing randomized controlled trials (RCTs), quality of life is often not set as the primary endpoint, and the assessment tools used are not standardized, leading to varied findings. Beck RW et al. (12) have evaluated quality of life using five scales, including the diabetes distress scale (DDS), and have found no significant differences between CGM and SMBG across all measures. In contrast, the multicenter open-label RCT reported by Thomas Haak et al. has revealed a substantial improvement in DDS in the CGM group in comparison with the SMBG group (13).

This review focuses specifically on emotional and patient-reported outcomes in adults with type 2 diabetes. This meta-analysis was performed to further systematically evaluate the influence of CGM on the quality of life of individuals with diabetes. This research integrated diverse quality-of-life assessment tools used across multiple RCTs, including DDS, diabetes treatment satisfaction questionnaire (DTSQ), world health organization-5 well-being index (WHO-5), and EuroQol five-dimensional questionnaire (EQ-5D). This multi-dimensional approach aims to comprehensively examine the potential role of CGM in improving patients’ quality of life and treatment experience, thereby providing more comprehensive evidence for clinical practice.

## 2 Methods

This research was conducted according to the Preferred Reporting Items for Systematic Reviews and Meta-Analyses (PRISMA) statement (14). The protocol was prospectively registered in the International Prospective Register of Systematic Reviews (PROSPERO) (registration number: CRD42025634725).

### 2.1 Literature search strategy

PubMed, Web of Science, Embase, and Cochrane Library were retrieved from their inception until April 2026. The search strategy combined terms for “type 2 diabetes” and “continuous glucose monitoring” using both medical subject headings (MeSH) and free-text terms. We explicitly excluded terms related to “Type 1 diabetes mellitus” from the search strategy using the NOT operator to ensure specificity. No language restrictions were applied during the initial search. The full, reproducible search strategies for all databases are provided in **Supplementary File S1**. Furthermore, the reference lists of included studies were manually screened to determine additional relevant literature.

### 2.2 Inclusion and exclusion criteria

The inclusion and exclusion criteria were formulated according to the population, intervention, comparator, outcomes, and study design (PICOS) framework. RCTs meeting the following criteria were included: the study population consisted of adult patients (≥18 years old) with type 2 diabetes. The intervention involved the use of CGM (e.g., rtCGM or isCGM), while the control group employed SMBG. The primary outcome was patients’ quality of life evaluated using validated scales (e.g., DDS, DTSQ, WHO-5, or EQ-5D). Exclusion criteria comprised: non-RCTs (e.g., reviews, systematic reviews and meta-analyses, observational studies), animal experiments, studies with non-extractable data, conference abstracts, and literature with full texts unavailable.

### 2.3 Literature screening and data extraction

Literature screening was independently performed by two researchers (Haibo Zhang and Jiebin Li). Initially, duplicate records were eliminated utilizing EndNote. Subsequently, articles were preliminary screened against titles and abstracts to remove obviously irrelevant literature. Finally, full texts of publications were evaluated to determine eligible studies. Any divergences were addressed through consultation with a third researcher (Jing Zhang).

Information was sourced from the included studies with a pre-designed standardized data extraction form. The acquired data encompassed: first author, publication year, country, sample size, baseline characteristics of patients (e.g., age, type of diabetes), specific details of the intervention and control groups, study duration, quality of life assessment tools used, and the corresponding outcome data (e.g., means and standard deviations).

### 2.4 Quality assessment of included studies

Two researchers (Haibo Zhang and Jiebin Li) independently examined the methodological quality of the included RCTs using the Cochrane risk of bias assessment tool (RoB 2.0) (15). This assessment involved five domains: randomization process, deviations from intended interventions, missing outcome data, measurement of the outcome, and selection of the reported result. Each domain was judged as “low risk”, “high risk”, or “some concerns”. Any divergences were addressed through discussion or consultation with a third researcher (Jing Zhang).

### 2.5 Statistical analysis

Statistical analysis was performed utilizing Stata 16.0. For continuous variables, the standardized mean difference (SMD) and its 95% confidence interval (CI) were established as the effect measure. SMD was chosen because the included studies used different versions or scoring conventions of the same scales (e.g., DTSQ), requiring standardization to a uniform scale. For each outcome, post-intervention scores (means and standard deviations at the end of the follow-up period) were extracted and used to calculate the SMD. Change-from-baseline scores were not used in the primary meta-analyses to maintain consistency across studies and to facilitate clinical interpretation. Heterogeneity across studies was assessed leveraging the I² statistic. In the case of I² ≤ 50% and P ≥ 0.1, heterogeneity was acceptable, and a fixed-effects model was adopted for the pooled analysis. For I² > 50% or P < 0.1, substantial heterogeneity was observed, a random-effects model was applied, and potential sources of heterogeneity were examined. Quantitative synthesis was performed only for outcomes assessed by the same validated instrument in at least two studies. Consequently, meta-analyses were conducted for DDS, DTSQ, WHO-5, and EQ-5D data. Other patient-reported measures (e.g., DQoL, glucose monitoring experience questionnaire (GMEQ), GMSS, hypoglycemia fear survey-II (HFS-II)) were not pooled due to insufficient data and were described narratively. All subgroup analyses (by region, follow-up duration, and CGM type) were *post-hoc* and exploratory in nature. They were conducted to generate hypotheses for future research and should be interpreted with caution due to the limited number of studies in each subgroup. Sensitivity analysis was carried out by sequentially omitting individual studies to determine the robustness of the pooled results. Publication bias was evaluated with funnel plots and Egger’s tests if ten or more studies were incorporated. The significance level was set at α = 0.05.

## 3 Results

### 3.1 Screening

**Figure 1:**
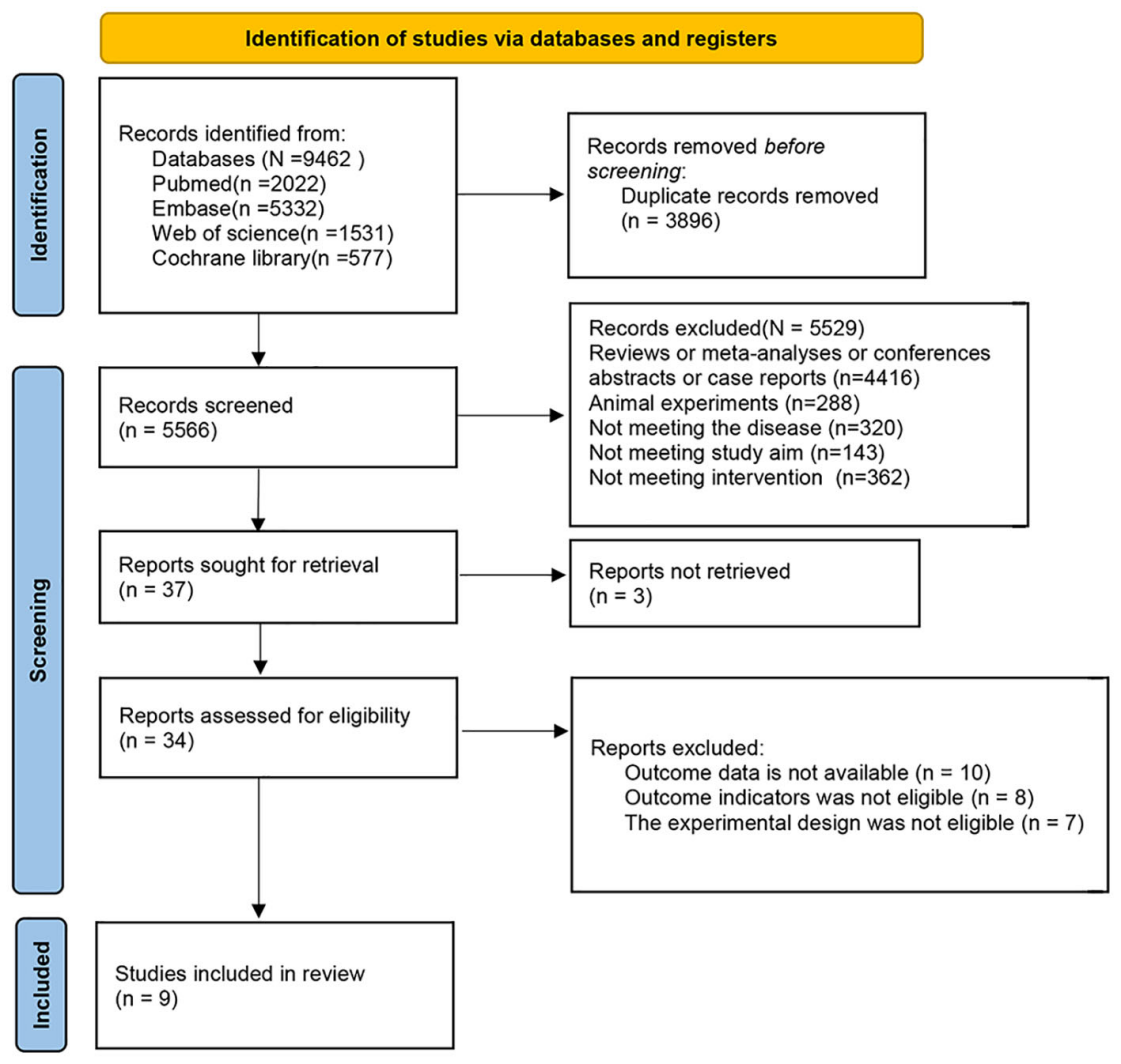
PRISMA flow diagram of study screening and selection.

A total of 9,462 records were initially identified. After removal of duplicates and exclusion of 5,566 records during initial screening, full contents of the remaining 34 articles were reviewed. Ultimately, 9 studies were incorporated in the analysis (**Figure 1**).

### 3.2 Characteristics

The nine studies included in this analysis were all RCTs (12, 13, 16–22), published between 2017 and 2025, with a total sample size of 1,453 individuals. The baseline characteristics of the included studies are presented in **Table 1**. These studies were conducted across multiple countries and regions, primarily in Europe, North America, Asia, and Australia. Sample sizes were from 76 to 299 individuals, and the mean age of individuals was from 52.8 to 61.2 years. In most studies, males were slightly more than females or the sex distribution was approximately balanced. Body mass index (BMI) varied substantially across studies, ranging from 26.8 to 36.1 kg/m². This result reflected certain heterogeneity in the weight status of the included populations.

**Table 1:** Characteristics of included studies.

ID	Author	Quality of life assessment tool	Year	Country	Sample size	Age	Sex (male/female)	BMI	Course of diabetes (years)	HbA1c%	Treatment	Blood glucose monitoring methods of the treatment group	Blood glucose monitoring methods of the control group	Follow-up time
2	Kim	DTSQ	2024	South Korea	159	58 (11)	85/74	27.1(4.7)	17.1(10.1)	8.4(1.0)	Insulin injection or pump	Personal, unblinded isCGM	SMBG	24 weeks
3	Haak	DTSQ, DQoL, DDS	2017	France, Germany, UK	224	59.2 (10.3)	150/74	33.2 (5.96)	17.3(8.0)	8.8(1.0)	Insulin injection	Personal, unblinded isCGM	SMBG	6 months
7	Speight	WHO-5, GMEQ	2021	Australia	299	60 (10)	176/123	N/A	12(8.2)	8.9(1.2)	Oral medication, insulin	Professional, blinded isCGM	SMBG	12 months
8	Chandran	EQ-5D	2024	Singapore	176	55 (10.7)	102/74	27.8 (5.9)	10.9(7.3)	8.4(0.6)	Oral medication, insulin	Personal, unblinded isCGM	SMBG	52 weeks
9	Lau	DTSQ	2024	Canada	105	57.3 ± 11.8	53/52	32.4 ± 7.2	<1 year: 13 people, 1–5 years: 18 people, 6–10 years: 32 people, > 10 years: 42 people	8.0(1.1)	Oral medication, insulin	Personal, unblinded isCGM	SMBG	6 weeks
12	Willis	DDS	2025	America	163	52.8 (9.5)	84/79	36.1 (7.6)	9.7 (7.7)	8.1 (1.2)	Oral medication, insulin	Personal, unblinded isCGM	SMBG	6 months
20	Wada	DTSQ	2020	Japan	93	58.4(9.9)	68/32	26.8(5.2)	N/A	7.8(0.3)	Oral medication, insulin	Personal, unblinded isCGM	SMBG	24 weeks
25	Lind	WHO-5, DDS, HFS-II, GMSS, DTSQ	2024	Denmark	76	61.2 (8.3)	32/44	31.7 (6.6)	18.1(6.8)	8.4(1.0)	Insulin injection	Personal, unblinded rtCGM	SMBG	12 months
28	Beck	DDS, EQ-5D, WHO-5	2017	North America	158	60 (10)	69/89	36(7.6)	17.3(8.7)	8.5(0.6)	Insulin injection	Personal, unblinded rtCGM	SMBG	24 weeks

DDS, diabetes distress scale; DTSQ, diabetes treatment satisfaction questionnaire; DQoL, diabetes quality of life; WHO−5, world health organization-5 well−being index; GMEQ, glucose monitoring experience questionnaire; EQ-5D, EuroQol five-dimensional questionnaire; HFS-II, hypoglycemia fear survey-II; GMSS, glucose monitoring satisfaction survey; N/A, not applicable; CGM, continuous glucose monitoring; isCGM, intermittently scanned CGM; rtCGM, real-time continuous glucose monitoring; SMBG, self-monitoring of blood glucose.

The majority of individuals had a long duration of diabetes, and averages ranged from 9.7 to 18.1 years. Only one study (Lau et al., 2024) included newly diagnosed patients with a disease duration of less than one year. Baseline HbA1c levels varied between 7.8% and 8.9%. These data uncovered generally suboptimal glycemic control among the included patients.

In terms of glucose monitoring technology, seven studies employed isCGM, while only two studies used rtCGM. This predominance of isCGM, which requires active scanning rather than automatic alerts, was a potential source of variability in treatment satisfaction outcomes. Regarding treatment modality, four studies exclusively enrolled participants receiving insulin therapy (multiple daily injections or insulin pump therapy), two studies included only non-insulin users, and the remaining three studies did not restrict enrollment based on insulin use. The intensity of insulin therapy might influence both glycemic and psychosocial outcomes. Follow-up duration varied considerably, ranging from 6 weeks to 52 weeks, and the majority of studies assessed outcomes at six months or less. This temporal variation might account for some of the dynamic changes in treatment satisfaction observed across different follow-up periods. Baseline HbA1c levels ranged from 7.8% to 8.9%, indicating generally suboptimal glycemic control across the included populations. Differences in baseline glycemic status might contribute to the heterogeneity in treatment responses.

### 3.3 Risk of bias assessment

**Figure 2:**
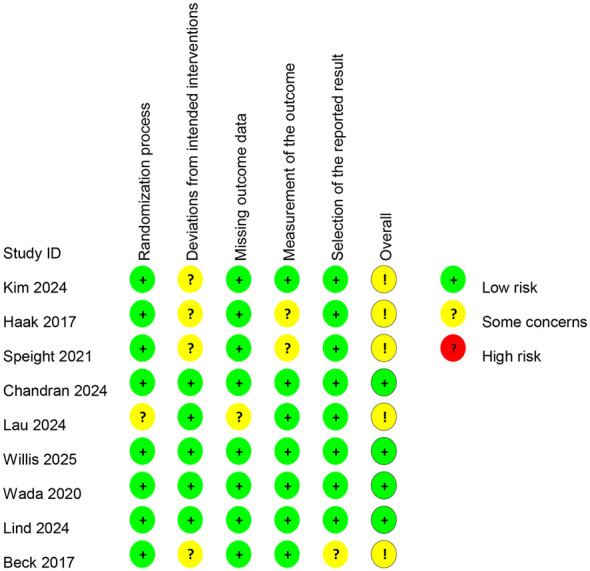
Risk of bias for studies.

The quality of the included studies was evaluated using the Cochrane RoB 2.0 tool. The results indicated that most domains were at low risk of bias, although some concerns were noted in certain areas, as illustrated in **Figure 2**.

Specifically, in the domain of the “randomization process,” eight studies were judged as low risk, while one study was rated as having “some concerns.” For “deviations from intended interventions,” five studies were at low risk, and four studies had “some concerns” due to the open-label nature of the CGM intervention, which precluded blinding of participants and investigators. In the domain of “missing outcome data,” eight studies were at low risk, and one study was rated as having “some concerns.” Regarding “measurement of the outcome,” seven studies were judged as low risk, and two studies had “some concerns.” For “selection of the reported result,” eight studies were at low risk, and one study was rated as having “some concerns”.

Although blinding was impractical due to the interventional nature of CGM, the primary outcomes (quality of life reported by patients) were assessed using validated standardized scales, and the data collection process was unlikely to be substantially influenced by knowledge of group allocation. Moreover, most studies had low rates of dropout and employed intention-to-treat analysis, effectively reducing the risk of attrition bias. Overall, the risk-of-bias profile supports the reliability of the pooled findings, despite the inherent methodological limitations of open-label behavioral interventions.

### 3.4 Outcomes

#### 3.4.1 DDS

**Figure 3:**
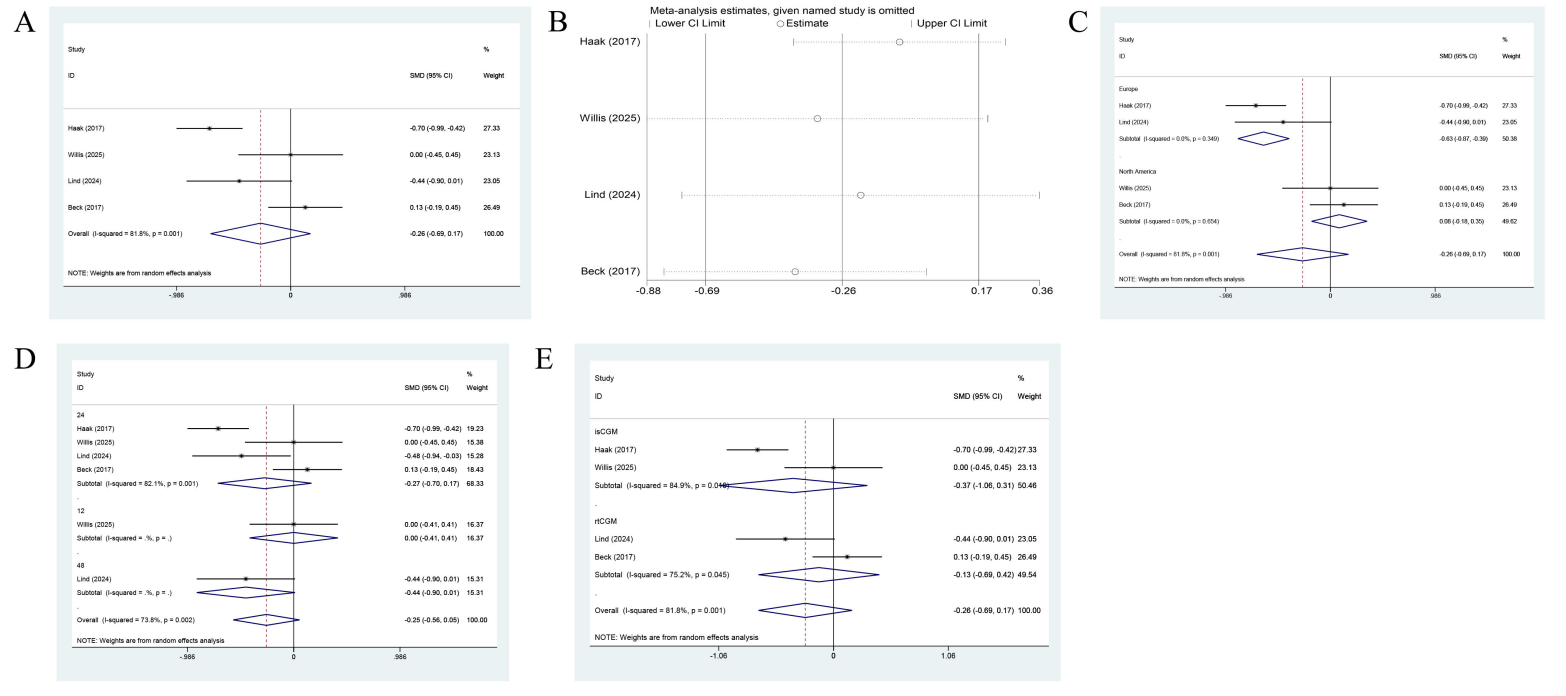
Forest plot for DDS outcomes. **(A)** Main pooled analysis comparing CGM with SMBG. **(B)** Leave-one-out sensitivity analysis assessing the influence of individual studies on the pooled estimate. **(C)** Exploratory subgroup analysis by geographic region. **(D)** Exploratory subgroup analysis by follow-up duration. **(E)** Exploratory subgroup analysis by CGM type (isCGM vs. rtCGM). Effect sizes are presented as SMDs with 95% CIs. Negative SMD values indicate a reduction in diabetes distress favoring CGM. Heterogeneity is assessed using the I² statistic.

A pooled analysis of four studies demonstrated that the relation between CGM and diabetes distress did not achieve statistical significance (pooled SMD = -0.25, 95% CI: -0.98 to 0.17, p > 0.05) (12, 13, 18, 22). Nevertheless, substantial heterogeneity was found across studies (I² = 81.8%, p = 0.001), as shown in **Figure 3**.

Sensitivity analysis was performed with the leave-one-out method to evaluate the robustness of the pooled results. After any single study among Haak (2017), Willis (2025), Lind (2024), or Beck (2017) was sequentially omitted, the recalculated pooled effect sizes (SMD) ranged from -0.41 to -0.08. The 95% CI consistently included zero, and none of the results achieved statistical significance. The main findings were robust.

Subgroup analyses were carried out to examine the sources of heterogeneity. In the subgroups stratified by region, CGM substantially reduced diabetes distress among European populations (SMD = -0.63, 95% CI: -0.97 to -0.39), and no heterogeneity was observed (I² = 0.0%). Notably, this subgroup analysis was based on a limited number of studies (n=2 in the European subgroup) and should be considered hypothesis-generating only. In contrast, no significant effect was found among North American populations (SMD = 0.08, 95% CI: -0.18 to 0.35). This result suggested that region may be an important factor for heterogeneity. Subgroup analysis by follow-up duration showed that neither the 24-week (SMD = -0.27, 95% CI: -0.70 to 0.17) nor the 48-week (SMD = -0.44, 95% CI: -0.90 to 0.01) subgroup yielded significant effects of CGM on diabetes distress, and higher heterogeneity persisted within each follow-up subgroup. These data indicated that follow-up duration was not a major source of heterogeneity. In the subgroups stratified by type of glucose monitoring, neither isCGM (SMD = -0.37, 95% CI: -1.06 to 0.31) nor rtCGM (SMD = -0.13, 95% CI: -0.88 to 0.42) showed significant effects, and moderate to high heterogeneity remained within each subgroup.

#### 3.4.2 DTSQ

**Figure 4:**
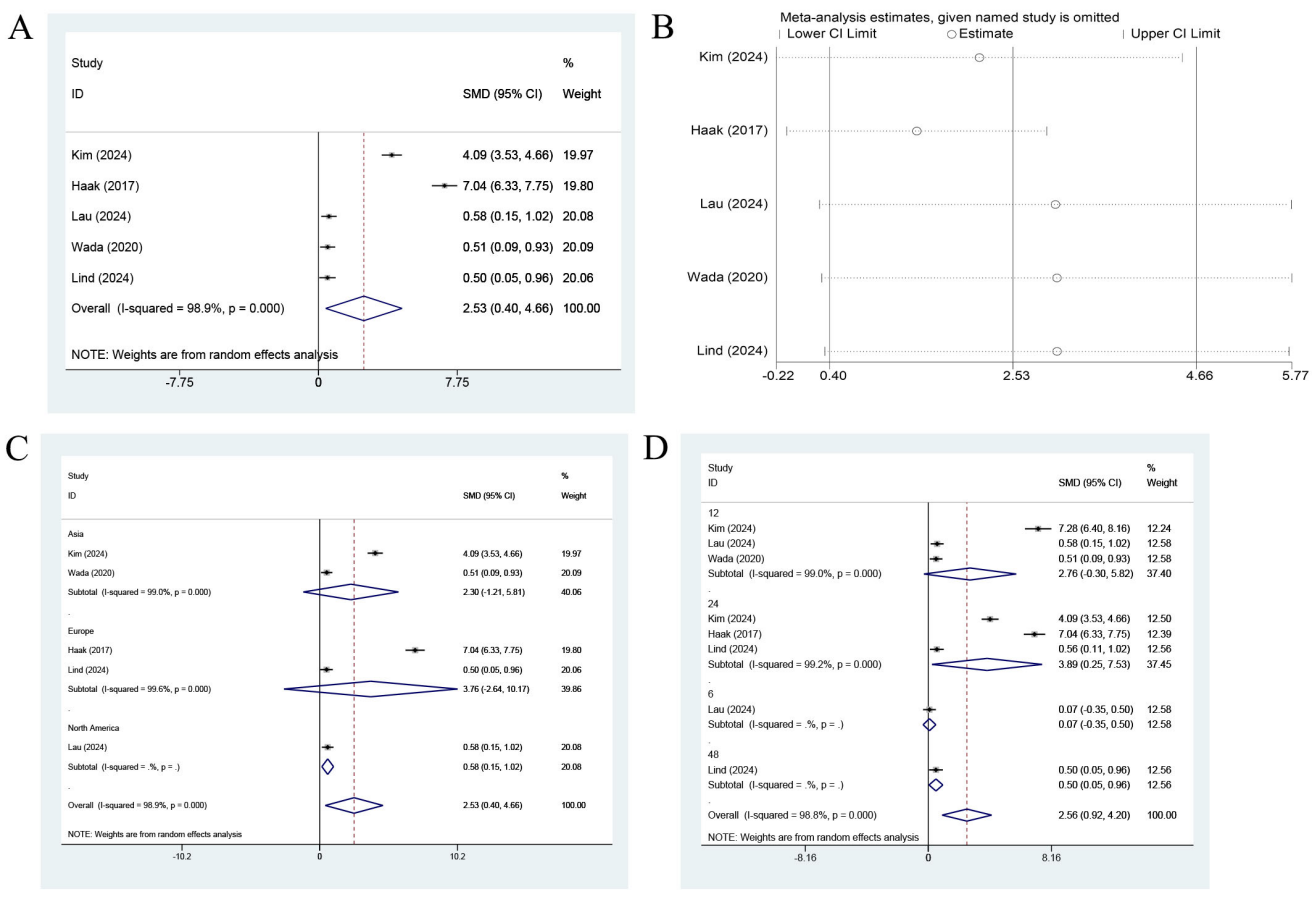
Forest plot for DTSQ outcomes. **(A)** Main pooled analysis comparing CGM with SMBG. Due to extremely high heterogeneity (I² = 98.9%), this pooled estimate should be interpreted with caution and is presented for transparency only. **(B)** Leave-one-out sensitivity analysis. **(C)** Exploratory subgroup analysis by geographic region. **(D)** Exploratory subgroup analysis by follow-up duration. Effect sizes are presented as SMDs with 95% CIs. Positive SMD values indicate higher treatment satisfaction favoring CGM.

Five studies reported DTSQ outcomes. Due to extremely high heterogeneity (I² = 98.9%, p < 0.001), a single pooled estimate was not clinically interpretable and was therefore not presented as the primary finding. All five studies showed a direction of effect favoring CGM over SMBG, but the magnitude of improvement varied substantially (SMDs ranging from 0.07 to 4.66 across studies). Given this inconsistency, the results were presented as a narrative synthesis, with the forest plot retained for transparency (**Figure 4A**).

Sensitivity analysis was performed on the pooled DTSQ results using the leave-one-out method to determine their robustness. After any single study among Kim (2024), Haak (2017), Lau (2024), Wada (2020), or Lind (2024) was sequentially omitted, the recalculated pooled effect sizes (SMD) varied between 1.41 and 3.04. The 95% CIs excluded zero and all results remained statistically substantial. Although some variations in point estimates were identified, the findings of all sensitivity analyses were directionally consistent with the initial pooled results (SMD = 2.53, 95% CI: 0.40 to 4.66) and they consistently demonstrated that CGM markedly improved treatment satisfaction (**Figure 4B**). While the direction of effect remained consistent when individual studies were omitted, the magnitude of the effect varied widely (SMDs ranging from 1.41 to 3.04). Given the extreme heterogeneity (I² = 98.9%), the robustness of a single summary estimate remained questionable, and the pooled result should not be overinterpreted.

Exploratory subgroup analyses were conducted to identify the sources of heterogeneity. For subgroup analysis by region, no statistically significant effects were identified in the European (SMD = 3.76, 95% CI: -2.64 to 10.17) or Asian (SMD = 2.30, 95% CI: -1.21 to 5.81) subgroup, whereas CGM significantly improved treatment satisfaction among individuals in North America (SMD = 0.58, 95% CI: 0.15 to 1.02). Notably, very high heterogeneity persisted within both the Asian and European subgroups (I² > 99.0%). This result suggested that regional differences only partially explained the overall heterogeneity. Given the small number of studies in each subgroup and the persistence of extreme heterogeneity, these regional differences should be interpreted with caution and considered hypothesis-generating only.

Subgroup analysis by follow-up duration revealed significant positive effects at 24 weeks (SMD = 3.89, 95% CI: 0.25 to 7.53), while smaller or non-significant effect sizes were identified at 6 weeks (SMD = 0.07, 95% CI: -0.35 to 0.50), 12 weeks (SMD = 2.76, 95% CI: -0.30 to 5.82) and 48 weeks (SMD = 0.50, 95% CI: 0.05 to 0.96). These findings suggested that treatment satisfaction might vary dynamically over the course of the intervention, but the small number of studies at each time point and the persistent high heterogeneity precluded any definitive conclusions regarding the optimal duration of CGM use for maximizing treatment satisfaction.

In summary, the available evidence consistently indicated that CGM use was associated with higher treatment satisfaction compared with SMBG in adults with type 2 diabetes. However, the magnitude of this benefit was highly variable across studies, likely reflecting differences in study populations, healthcare contexts, CGM device types, and concomitant educational interventions. The extreme heterogeneity observed (I² = 98.9%) precluded a meaningful single summary estimate, and the exploratory subgroup analyses, while suggestive of potential regional and temporal patterns, were based on very limited data. Consequently, although the direction of effect consistently favored CGM, the precise magnitude of improvement in treatment satisfaction attributable to CGM remained uncertain and likely context-dependent.

#### 3.4.3 WHO-5

A pooled analysis of only three studies (n=533 participants) showed that the improvement in psychological well-being among individuals with diabetes by CGM did not achieve statistical significance (pooled SMD = 0.08, 95% CI: -0.09 to 0.25, p > 0.05) (12, 18, 20). The limited number of studies constrained the certainty and generalizability of this finding. This finding suggested that, in comparison with the control group, CGM may be associated with a slight positive effect, but the effect size was minimal and accompanied by substantial uncertainty.

Heterogeneity across the studies was low (I² = 0.0%, p = 0.375), indicating good consistency across the findings. Nevertheless, the point estimates of the three included studies (Speight 2021; Lind 2024; Beck 2017) were 0.05, 0.38, and 0.00, respectively. They all approached the null line, and their 95% CIs were either wide or included zero, further supporting the robustness of the conclusion of no significant effect.

**Figure 5:**
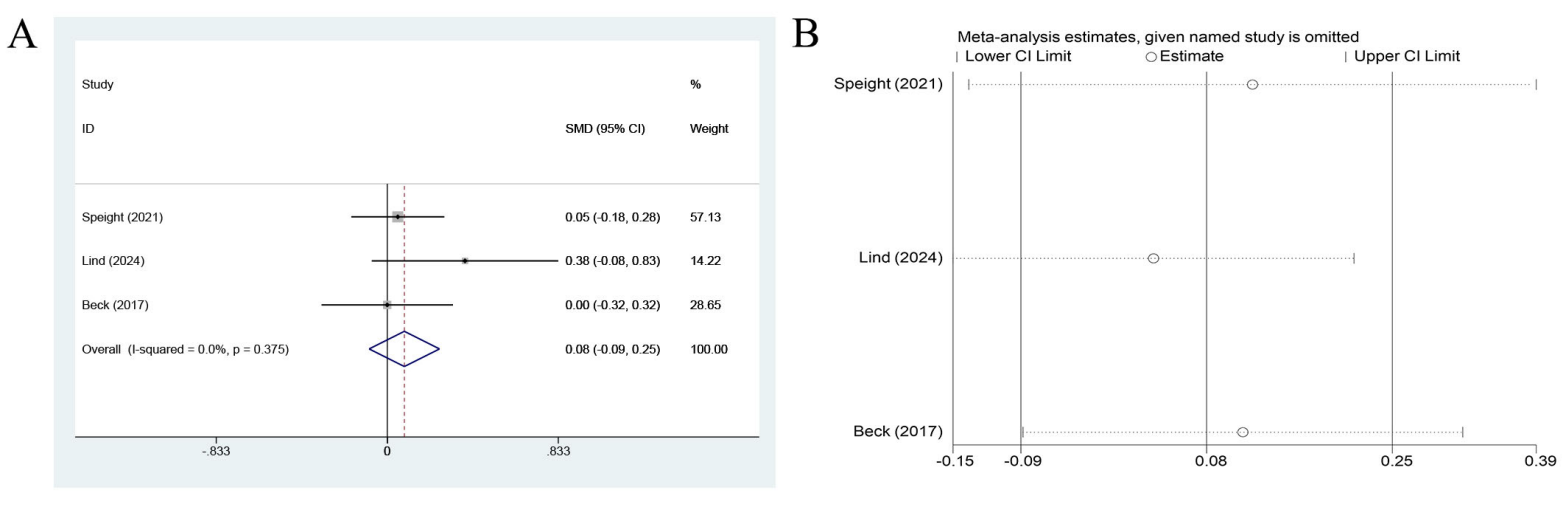
Forest plot for WHO-5 outcomes. **(A)** Main pooled analysis comparing CGM with SMBG based on three studies. **(B)** Leave-one-out sensitivity analysis. Effect sizes are presented as SMDs with 95% CIs. Positive SMD values indicate better psychological well-being favoring CGM. Low heterogeneity (I² = 0.0%) was observed across studies.

Sensitivity analysis was performed on the pooled WHO-5 results using the leave-one-out method to determine their robustness. As illustrated in **Figure 5**, after any single study among Speight (2021), Lind (2024), or Beck (2017) was sequentially omitted, the recalculated pooled effect sizes (SMD) ranged from 0.034 to 0.154. Their 95% CIs all included zero and all results did not reach statistical significance. These findings were fully consistent with the initial pooled result (SMD = 0.083, 95% CI: -0.088 to 0.255). The effect sizes varied within a very narrow range across the analyses, further confirming the high stability of the primary conclusion. Based on the previously observed low heterogeneity (I² = 0.0%), the finding of CGM with no significant effect on patients’ psychological well-being was reliable and robust.

#### 3.4.4 EQ-5D

**Figure 6:**
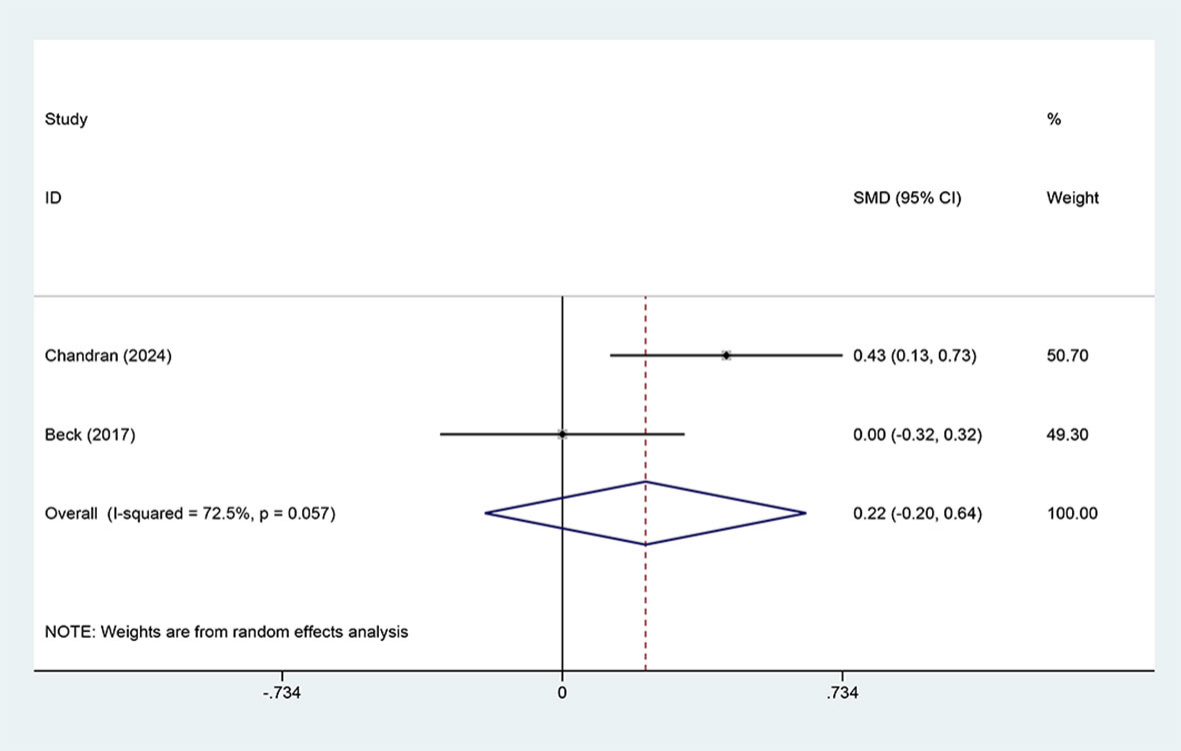
Forest plot for EQ-5D outcomes. Pooled analysis comparing CGM with SMBG based on two studies. Effect sizes are presented as SMDs with 95% CIs. Positive SMD values indicate improved health-related quality of life favoring CGM, although the pooled estimate was not statistically significant and moderate-to-high heterogeneity was present (I² = 72.5%).

A pooled analysis of only two studies (n=334 participants) investigated the effect of CGM on health-related quality of life in individuals with diabetes (12, 19). This severely limited the statistical power and generalizability of the analysis. The random-effects model generated a pooled effect size (SMD) of 0.22 (95% CI: -0.20 to 0.64), which did not achieve statistical significance (p > 0.05) (**Figure 6**). The finding uncovered that, compared with the control group, CGM may be associated with a small positive trend toward improved quality of life, but this effect remained unclear.

However, substantial heterogeneity was identified between the two studies (I² = 72.5%, p = 0.057). This result may be attributable to differences in characteristics of the study population, regions, or specific intervention protocols. Specifically, Chandran (2024) reported a significant moderate effect improvement of CGM (SMD = 0.43, 95% CI: 0.13 to 0.73), whereas Beck (2017) demonstrated no effect (SMD = 0.00, 95% CI: -0.32 to 0.32). This discrepancy in findings largely accounted for the non-significant pooled effect and the substantial heterogeneity observed.

## 4 Discussion

This research pooled data from nine RCTs comprising 1,453 individuals with type 2 diabetes. It intended to evaluate the multi-dimensional impact of CGM on patients’ emotional well-being. The findings suggest that CGM may improve treatment satisfaction (DTSQ), although this conclusion is limited by extreme heterogeneity. However, CGM showed no significant advantages in alleviating diabetes distress (DDS), enhancing psychological well-being (WHO-5), or improving health-related quality of life (EQ-5D). It is crucial to distinguish treatment satisfaction, a measure of the patient’s experience with the monitoring method itself, from broader emotional well-being constructs such as diabetes distress and general psychological health. Our findings indicate that while patients may find CGM more convenient and acceptable, this does not necessarily translate into alleviation of the deeper, multifactorial psychosocial burdens associated with living with type 2 diabetes. A particularly noteworthy finding from our subgroup analysis is the significant reduction in diabetes distress observed among European participants (SMD = -0.63, 95% CI: -0.97 to -0.39, I²=0%), a benefit not seen in North American cohorts. Although this finding is exploratory due to the small number of studies (n=2 in the European subgroup), the discrepancy warrants careful consideration. It may reflect fundamental differences in how CGM is integrated into clinical care pathways. In many European centers, CGM initiation is often coupled with structured diabetes education and ongoing psychosocial support, whereas North American models may treat the technology more as a stand-alone tool for glycemic management. Furthermore, variations in healthcare access, patient-provider communication styles, and cultural perceptions of chronic disease self-management could modulate the emotional impact of using CGM. This finding should be prioritized in future research.

Prior meta-analyses have primarily focused on the advantages of CGM in glycemic control metrics like HbA1c and TIR. However, systematic reviews addressing its impact on emotional well-being and quality of life remain relatively limited. The findings of this research are consistent with several earlier investigations. For instance, Beck et al. have similarly reported no significant improvements in diabetes distress or overall quality of life with CGM (12). Other studies, like a study by Haak et al., have documented positive effects of CGM on diabetes distress. Such discrepancies may be attributable to variations in characteristics of the study population, follow-up duration, assessment instruments, and CGM types (isCGM and rtCGM) (13). The results of the present research are highly consistent with those of a meta-analysis by Ferreira et al. for non-insulin-treated type 2 diabetes patients with respect to treatment satisfaction. Research by Ferreira et al. has also reported that CGM can significantly enhance treatment satisfaction. Nevertheless, it does not encompass other emotional well-being dimensions like diabetes distress or psychological well-being. This result suggests that the majority of investigations at present emphasize glycemic indicators and treatment satisfaction (9). Conversely, a meta-analysis by Klak et al. for type 1 diabetes patients has demonstrated that CGM can significantly reduce fear of hypoglycemia but shows no significant effect on treatment satisfaction (23). This contrast with our findings in type 2 diabetes is instructive. In type 1 diabetes, the primary psychological burden is often centered on fear of severe hypoglycemia, which is effectively mitigated by CGM through alerts and trend data. In type 2 diabetes, however, diabetes distress is a more complex, multifactorial construct that a monitoring device alone cannot fully address, as it is often rooted in regimen burden, perceived treatment failure, comorbidity management, and broader psychosocial stressors. This discrepancy highlights that the psychosocial impact of CGM is likely specific to the type of diabetes. These data indicate that the influence of CGM on emotional well-being may vary with diabetes type, treatment modality, and the nature of psychological burden. Both studies have also acknowledged limitations including heterogeneity and inconsistency in assessment tools, further supporting the findings and discussions of this research. By integrating multiple assessment instruments, this research provides more comprehensive evidence supporting the positive effects of CGM in improving treatment satisfaction.

The observed improvement in treatment satisfaction may stem from several plausible mechanisms. By providing continuous and visualized glucose data, CGM may deliver immediate feedback and behavioral cues, potentially enhance patients’ sense of control and self-efficacy in disease management, and reduce the inconvenience associated with traditional fingertip blood collection. However, these mechanisms were not directly measured in the included studies and remain speculative (9). Haak et al. (13) have observed that participants in the intervention group perform significantly more daily sensor scans compared to those involving baseline fingertip glucose measurements. Over time, by using historical glucose curves and trend arrows, patients proactively adjusted the amount of bedtime snacks or basal insulin doses. This results in significantly reduced nocturnal hypoglycemia and fewer daytime hypoglycemic events. This closed-loop cycle of real-time visualization, behavioral adjustment, and immediate glycemic benefit reinforced self-efficacy, thereby directly improving DTSQ. Notably, in the same trial, no between-group difference in HbA1c was found. This finding suggests that the improvement in satisfaction is not driven by “improved glucose numbers,” but rather stems from a purely experiential mechanism of “reduced testing burden + enhanced sense of control.” Similarly, a study by Klak et al. (2021) (23) has demonstrated that CGM reduces scores of the hypoglycemia fear scale–worry subscale (HFS-W) and improves DTSQ. Nocturnal hypoglycemia alerts and trend arrows liberate patients from fear of hypoglycemia during sleep and exercise, restoring a sense of freedom in social and physical activity contexts. This sense of safety is identified as the primary driver of improvements in satisfaction. Furthermore, continuous glucose curves transform abstract “fluctuations” into visual materials that can be discussed together, thus shifting clinical follow-up from “didactic physician lecturing” to “shared decision-making through joint graph review.” Most participants have reported that adjusting insulin doses is easier with the aid of CGM devices and they are willing to continue using these devices beyond the study period. As a visual medium, CGM may enhance the quality of interactions between patients and clinicians during visits, facilitating collaborative optimization of medication dosing and lifestyle, thereby indirectly improving treatment confidence and satisfaction (10, 12). Notably, the effects of CGM may be amplified when CGM is combined with structured education. For instance, research by Lau et al. has revealed that in non-insulin-treated type 2 diabetes patients, CGM integrated with remote diabetes education not only significantly improves glycemic control but also enhances treatment satisfaction and promotes weight loss (17). This result suggests that CGM functions not merely as a monitoring tool, but, through visualized data feedback and educational engagement, may foster behavioral change and bolster self-management confidence, thus positively influencing the treatment experience. However, diabetes distress, psychological well-being, and overall quality of life (as measured by EQ-5D, WHO-5, and DDS) are influenced by multidimensional factors, including illness-related cognitive burden, social support, concerns about complications, and fear of hypoglycemia (23). Consequently, optimization of glucose monitoring technology alone may be insufficient to significantly alleviate these deeply-rooted multifaceted psychosocial burdens (12). Moreover, cultural background, health beliefs, and socioeconomic conditions may moderate the effects of CGM on emotional well-being. This observation is consistent with the regional heterogeneity identified in our subgroup analyses. For future mechanism studies, it is necessary to systematically integrate the interactive effects of psychological, behavioral, and socio-environmental factors.

Conversely, it is also important to acknowledge that continuous data streams may have unintended negative consequences for some individuals. Potential issues such as “alarm fatigue” or increased anxiety from constant glucose monitoring (“data overload”) may counteract some of the positive effects on well-being, particularly in patients with pre-existing diabetes distress. These factors may partially explain why benefits in treatment satisfaction do not consistently translate to improvements in broader measures like the DDS or WHO-5.

Although the efficacy of CGM in improving glycemic indicators has been well established, this research indicates that it also offers clear benefits in enhancing patients’ treatment experience and may serve as an adjunctive strategy to improve treatment adherence. In clinical practice, the use of CGM may be considered based on the needs of individual patients, particularly among those with low treatment satisfaction or resistance to conventional glucose monitoring methods. However, given the very high heterogeneity in treatment satisfaction effects and the lack of clear benefit in other psychosocial domains, recommendations for practice should remain modest and acknowledge the current uncertainty in the evidence base. However, for patients whose primary goals include alleviating diabetes distress or improving overall quality of life, CGM may need to be combined with other psychosocial interventions.

Several limitations of this research should be noted. First, although this meta-analysis strictly adhered to the PRISMA guidelines, substantial heterogeneity across the included studies remained, particularly for DTSQ and DDS. Subgroup analyses were carried out based on region, follow-up duration, and type of glucose monitoring. However, the sources of heterogeneity could not be fully elucidated. This result may be attributable to multiple factors, including differences in versions of assessment tools, heterogeneity in baseline characteristics of patients (e.g., wide range of diabetes duration, varying comorbidity profiles), and variations in CGM intervention protocols (e.g., isCGM and rtCGM device types, sensor wear duration). Second, the number of eligible RCTs was relatively limited, and only nine studies were ultimately included. Consequently, certain key outcomes (e.g., EQ-5D) were pooled based on as few as two studies. This limitation substantially constrains the statistical robustness and generalizability of the findings. Third, the available evidence predominantly consisted of short-term follow-up data (most ≤6 months). These data may be insufficient to capture the long-term trajectory of CGM’s effects on emotional well-being, particularly given that psychological adaptation to chronic conditions often requires a longer time window to become evident. Fourth, data for quality of life were highly dependent on self-reported scales. Although these instruments were rigorously validated, the potential for expectancy effects and social desirability bias cannot be entirely excluded and may be more pronounced in open-label designs where patients were aware of their group allocation. This inherent limitation is reflected in the RoB 2.0 assessment, where several studies were rated as having ‘some concerns’ in the domains of ‘deviations from intended interventions’ and ‘measurement of the outcome’. Finally, as a physical device and behavioral intervention, CGM precludes blinding of participants and investigators. Although the included studies were identified as having low risk of bias in blinding for the measurement of the outcome, knowledge of group allocation may still have a potential influence on certain subjective self-reported outcomes, reflecting an inherent methodological challenge in this field.

Large-scale multicenter RCTs with long-term follow-up should be performed in the future. Standardized, multi-dimensional, and validated assessment tools for emotional well-being and quality of life should be used to more comprehensively evaluate the psychosocial benefits of CGM. Additionally, further investigations should be carried out to examine the differential effects on emotional well-being by CGM type, frequency of use, and subgroups (e.g., diverse cultural backgrounds, disease duration, treatment regimens, health beliefs, and socioeconomic conditions), thereby providing more precise evidence for developing personalized glucose monitoring strategies in clinical practice.

## 5 Conclusion

This systematic review and meta-analysis found that CGM may improve treatment satisfaction in adults with type 2 diabetes, although this finding is tempered by very high heterogeneity (I² = 98.9%) in the effect size. Evidence regarding its effects in alleviating diabetes distress, enhancing psychological well-being, or improving health-related quality of life remains inconclusive. The psychological benefits of CGM appear to be domain-specific and potentially moderated by contextual factors such as healthcare system and culture, as suggested by exploratory subgroup analyses. Future large-scale, long-term trials with standardized psychosocial outcome assessments are required to confirm these findings and to identify which patient subgroups are most likely to experience meaningful improvements in their emotional well-being from CGM use.

## Data Availability

The original contributions presented in the study are included in the article/**Supplementary Material**. Further inquiries can be directed to the corresponding author.

## Supplementary material

Supplementary File S1 Search strategy.
