# Supplementary material for: Impact of continuous glucose monitoring on patient-reported outcomes in adults with type 2 diabetes: a Systematic Review and meta-analysis
Source: Front Endocrinol (Lausanne). 2026 May 20;17:1830980. doi: 10.3389/fendo.2026.1830980 (PMC13229700; doi:10.3389/fendo.2026.1830980)
Supplement: Supplementary File S1 — Search strategy. [file Table1.docx]

**File S1** Search strategy

| Pubmed | |
| --- | --- |
| # | Query |
| 1 | Diabetes Mellitus, Type 2[MeSH Terms] |
| 2 | "adult onset diabetes"[Title/Abstract] OR "diabetes mellitus type 2"[Title/Abstract] OR "diabetes mellitus type ii"[Title/Abstract] OR "diabetes type 2"[Title/Abstract] OR "diabetes type II"[Title/Abstract] OR "dm 2"[Title/Abstract] OR "insulin independent diabetes"[Title/Abstract] OR "Ketosis Resistant Diabetes Mellitus"[Title/Abstract] OR "Maturity Onset Diabetes"[Title/Abstract] OR "MODY"[Title/Abstract] OR "NIDDM"[Title/Abstract] OR "non insulin dependent diabetes"[Title/Abstract] OR "noninsulin dependent diabetes"[Title/Abstract] OR "Slow Onset Diabetes Mellitus"[Title/Abstract] OR "Stable Diabetes Mellitus"[Title/Abstract] OR "T2DM"[Title/Abstract] OR "TIIDM"[Title/Abstract] OR "type 2 diabetes"[Title/Abstract] OR "type 2 insulin independent diabetes"[Title/Abstract] OR "type II diabetes"[Title/Abstract] |
| 3 | #1 OR #2 |
| 4 | Continuous Glucose Monitoring[MeSH Terms] |
| 5 | "CGM Device*"[Title/Abstract] OR "continuous glucose monitor technology"[Title/Abstract] OR "continuous glucose monitoring"[Title/Abstract] OR "continuous monitoring of blood glucose"[Title/Abstract] OR "FGM"[Title/Abstract] OR "flash glucose monitoring"[Title/Abstract] OR "freestyle libre"[Title/Abstract] OR "intermittent scanning continuous glucose monitoring"[Title/Abstract] OR "intermittently scanned continuous glucose monitoring"[Title/Abstract] OR "intermittent-scanned continuous glucose monitoring"[Title/Abstract] OR "isCGM"[Title/Abstract] |
| 6 | #4 OR #5 |
| 7 | #3 AND #6 |
| 8 | "Diabetes Mellitus, Type 1"[Mesh] OR "type 1 diabet*"[Title/Abstract] OR "T1D"[Title/Abstract] OR "T1DM"[Title/Abstract] OR "IDDM"[Title/Abstract] |
| 9 | #7 NOT #8 |

| Embase | |
| --- | --- |
| # | Query |
| 1 | 'non insulin dependent diabetes mellitus'/exp |
| 2 | 'adult onset diabetes':ti,ab,kw OR 'diabetes mellitus type 2':ti,ab,kw OR 'diabetes mellitus type ii':ti,ab,kw OR 'diabetes type 2':ti,ab,kw OR 'diabetes type ii':ti,ab,kw OR 'dm 2':ti,ab,kw OR 'insulin independent diabetes':ti,ab,kw OR 'ketosis resistant diabetes mellitus':ti,ab,kw OR 'maturity onset diabetes':ti,ab,kw OR 'mody':ti,ab,kw OR 'niddm':ti,ab,kw OR 'non insulin dependent diabetes':ti,ab,kw OR 'noninsulin dependent diabetes':ti,ab,kw OR 'slow onset diabetes mellitus':ti,ab,kw OR 'stable diabetes mellitus':ti,ab,kw OR 't2dm':ti,ab,kw OR 'tiidm':ti,ab,kw OR 'type 2 diabetes':ti,ab,kw OR 'type 2 insulin independent diabetes':ti,ab,kw OR 'type ii diabetes':ti,ab,kw |
| 3 | #1 OR #2 |
| 4 | 'continuous glucose monitoring'/exp |
| 5 | 'cgm device*':ti,ab,kw OR 'continuous glucose monitor technology':ti,ab,kw OR 'continuous glucose monitoring':ti,ab,kw OR 'continuous monitoring of blood glucose':ti,ab,kw OR 'fgm':ti,ab,kw OR 'flash glucose monitoring':ti,ab,kw OR 'freestyle libre':ti,ab,kw OR 'intermittent scanning continuous glucose monitoring':ti,ab,kw OR 'intermittently scanned continuous glucose monitoring':ti,ab,kw OR 'intermittent-scanned continuous glucose monitoring':ti,ab,kw OR 'iscgm':ti,ab,kw |
| 6 | #4 OR #5 |
| 7 | #3 AND #6 |
| 8 | ‘insulin dependent diabetes mellitus’/exp |
| 9 | ‘type 1 diabet*’:ti,ab,kw OR ‘T1D’:ti,ab,kw OR ‘T1DM’:ti,ab,kw OR ‘IDDM’:ti,ab,kw |
| 10 | #8 OR #9 |
| 11 | #7 NOT #10 |

| Cochrane Library | |
| --- | --- |
| # | Query |
| 1 | MeSH descriptor: [Diabetes Mellitus, Type 2] explode all trees |
| 2 | ('adult onset diabetes' OR 'diabetes mellitus type 2' OR 'diabetes mellitus type ii' OR 'diabetes type 2' OR 'diabetes type II' OR 'dm 2' OR 'insulin independent diabetes' OR 'Ketosis Resistant Diabetes Mellitus' OR 'Maturity Onset Diabetes' OR 'MODY' OR 'NIDDM' OR 'non insulin dependent diabetes' OR 'noninsulin dependent diabetes' OR 'Slow Onset Diabetes Mellitus' OR 'Stable Diabetes Mellitus' OR 'T2DM' OR 'TIIDM' OR 'type 2 diabetes' OR 'type 2 insulin independent diabetes' OR 'type II diabetes'):ti,ab,kw |
| 3 | #1 OR #2 |
| 4 | MeSH descriptor: [Continuous Glucose Monitoring] explode all trees |
| 5 | ('CGM Device*' OR 'continuous glucose monitor technology' OR 'continuous glucose monitoring' OR 'continuous monitoring of blood glucose' OR 'FGM' OR 'flash glucose monitoring' OR 'freestyle libre' OR 'intermittent scanning continuous glucose monitoring' OR 'intermittently scanned continuous glucose monitoring' OR 'intermittent-scanned continuous glucose monitoring' OR 'isCGM'):ti,ab,kw |
| 6 | #4 OR #5 |
| 7 | #3 AND #6 |
| 8 | MeSH descriptor: [Diabetes Mellitus, Type 1] explode all trees |
| 9 | (‘type 1 diabet*’ OR ‘T1D’ OR ‘T1DM’ OR ‘IDDM’):ti,ab,kw |
| 10 | #8 OR #9 |
| 11 | #7 NOT #10 |

| Web of Science | |
| --- | --- |
| # | Query |
| 1 | TS=((adult onset diabetes) OR (diabetes mellitus type 2) OR (diabetes mellitus type ii) OR (diabetes type 2) OR (diabetes type II) OR (dm 2) OR (insulin independent diabetes) OR (Ketosis Resistant Diabetes Mellitus) OR (Maturity Onset Diabetes) OR (MODY) OR (NIDDM) OR (non insulin dependent diabetes) OR (noninsulin dependent diabetes) OR (Slow Onset Diabetes Mellitus) OR (Stable Diabetes Mellitus) OR (T2DM) OR (TIIDM) OR (type 2 diabetes) OR (type 2 insulin independent diabetes) OR (type II diabetes)) |
| 2 | TS=((CGM Device*) OR (continuous glucose monitor technology) OR (continuous glucose monitoring) OR (continuous monitoring of blood glucose) OR (FGM) OR (flash glucose monitoring) OR (freestyle libre) OR (intermittent scanning continuous glucose monitoring) OR (intermittently scanned continuous glucose monitoring) OR (intermittent-scanned continuous glucose monitoring) OR (isCGM)) |
| 3 | #2 AND #1 |
| 4 | TS=((type 1 diabet*) OR (T1D) OR (T1DM) OR (IDDM)) |
| 5 | #3 NOT #4 |
